# Supplementary figures and images for: Systemic complement activation levels in Stargardt disease
Source: PLoS One. 2021 Jun 25;16(6):e0253716. doi: 10.1371/journal.pone.0253716 (PMC8232401; doi:10.1371/journal.pone.0253716)

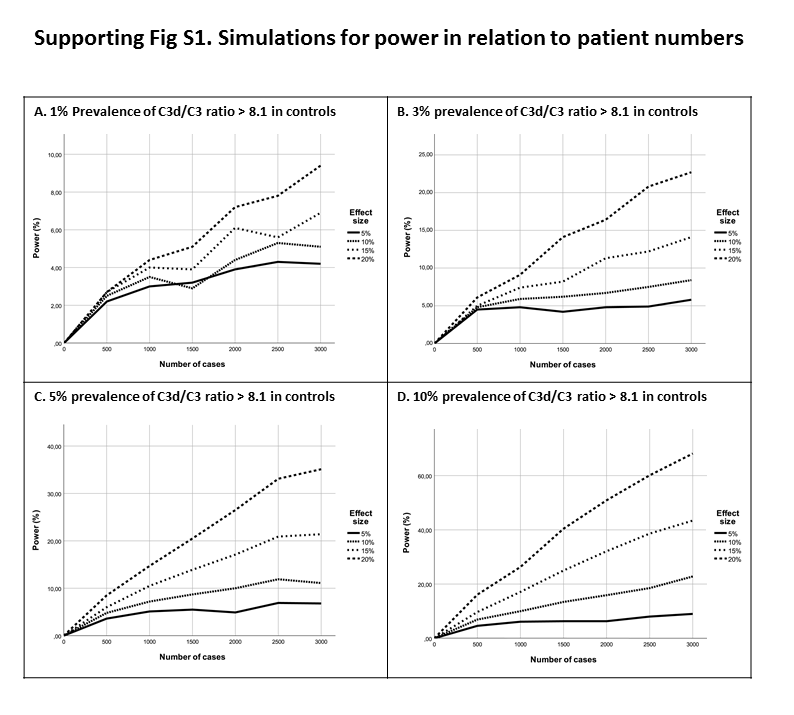

Supplement: S1 Fig — Panels display different prevalences of an above normal C3d/C3 ratio (>8.1) in controls; (A) 1%, (B) 3%, (C) 5%, (D) 10%. Graphs display the number of cases in relation to the statistical power of the study, assuming the 1:1 matched design of the current study. Each graph contains 4 lines with different effect sizes (5%, 10%, 15%, 20%). Very large cohorts are needed to detect small effect sizes. (TIF) [file pone.0253716.s001.tif]

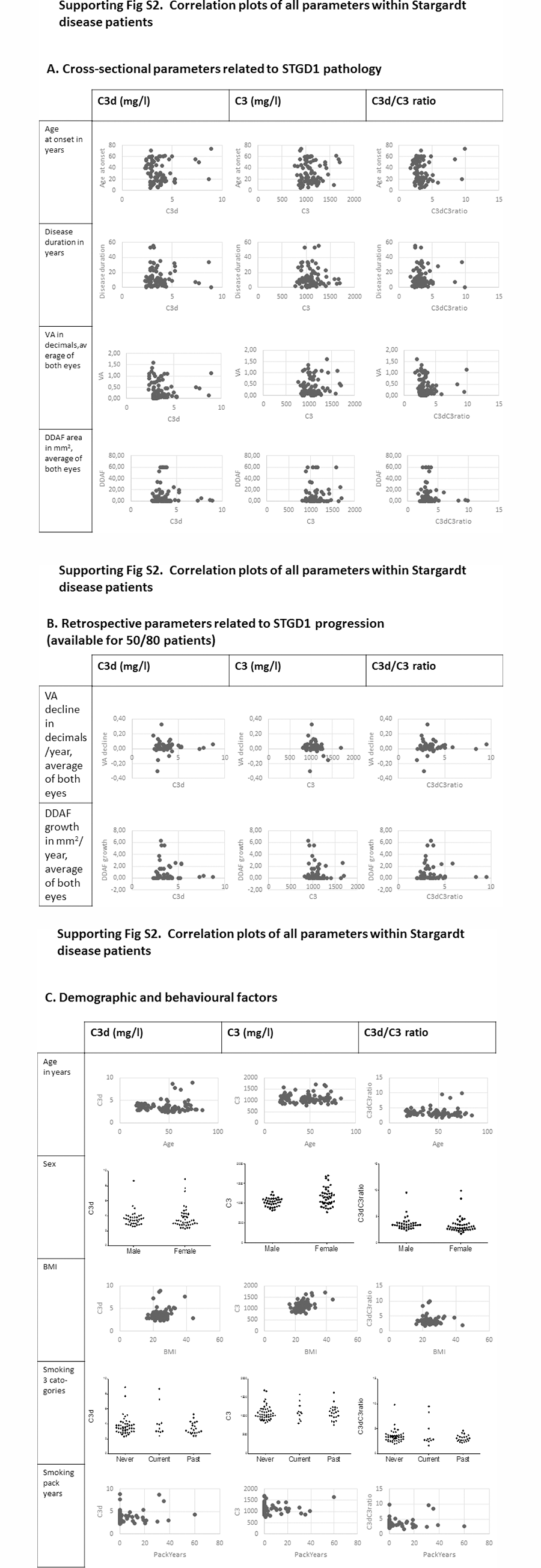

Supplement: S2 Fig — Correlation plots of respectively C3d, C3 and C3d/C3 ratio with (A) cross-sectional parameters related to STGD1 pathology, (B) retrospective parameters related to STGD1 progression and (C) demographic and behavioral factors. (TIF) [file pone.0253716.s002.tif]
